# Supplementary material for: Histological and immunohistochemical characterization of the porcine ocular surface
Source: PLoS One. 2020 Jan 13;15(1):e0227732. doi: 10.1371/journal.pone.0227732 (PMC6957219; doi:10.1371/journal.pone.0227732)
Supplement: Attachment — Submitted filename: Response to Reviewers.docx [file pone.0227732.s001.docx]

Yolanda Diebold, Ph.D.

IOBA-University of Valladolid

Paseo de Belén 17, 47011, Valladolid, Spain

December 20th, 2019

Dear Academic Editor Yu-Chi Liu, M.D

Please find attached the revised version of the manuscript PONE-D-19-32784 entitled: *“Histological and inmunohistochemical characterization of the porcine ocular surface”.* We have revised it and completed it according to reviewer’s requirements. Below we explain point by point the changes that we have made. We have uploaded to Figshare a document with raw data to reply Reviewer #2 Response to Question 3 (https://figshare.com/articles/Histological_and_immunohistochemical_characterization_of_the_porcine_ocular_surface_Raw_data_xlsx/11417130). Modifications in the manuscript are shown with track changes in order to identify them easily.

Thank you very much for giving us the opportunity to revise our manuscript. We hope that this revised version is now suitable for publication in *Plos One.*

Thank you very much for your consideration,

Sincerely,

Yolanda Diebold, Ph.D.

**RESPONSE TO THE JOURNAL REQUIEREMENTS AND THE SUGGESTIONS OF THE REVIEWERS**

Journal Requirements:

Thank you very much for remembering us to accomplish these Journal Requirements.

1. *Please ensure that your manuscript meets PLOS ONE’s style requirements, including those for file naming.*

We have made the necessary changes in the manuscript to be sure that it follows PLOS ONE’s submission guidelines.

2. At this time, we ask that you please provide the source and any product numbers and lot numbers of the lectins PNA and HPA used in your study in the Methods section of the manuscript.

We have included this information in the Methods section of the manuscript as required.

Reviewer #1:

Thank you very much for your useful comments. As suggested, we have included the negative control in Figure 11. Below we respond to all of your specific questions.

1. *In line 105, pre-adult pigs (6-8 weeks) were used. Are there any specific reasons to use pre-adult pigs? Will the ocular surface characteristics be different when pigs become adult?*

The reason to use animals in this age (6-8 months, not weeks) is that we use pigs that are designated to human consumption, and that is the age at which they go to the slaughterhouse. We have not found any information in the literature suggesting that the ocular surface characteristics may change after this age. These animals are close to or into their sexual maturation period. We consider that no bigger differences would be found between them and young adult pigs.

2. *Figure legends should not be inserted into the main text.*

The *Plos One* Figure instructions demands to “*place figure captions in the manuscript text in read order, immediately following the paragraph where the figure is first cited. Do not include captions as part of the figure files or submit them in a separate document.”*

3. *Should include a negative control in Figure 11.*

We have included the negative control of the CD3 inmunofluorescence keeping the structure of the figure: Blue channel (nucleus), green channel (CD3 positive), merge.

Reviewer #2:

Thank you very much for your useful comments and suggestions. Below we respond to all of your specific comments. Changes in the manuscript can be seen in the track changes version.

1. *One of the challenges of using pig tissues for research is the limited number of antibodies that recognize porcine antigens. This perhaps should be highlighted in the discussion.*

We completely agree with this affirmation and we have now added a sentence in the discussion including this statement.

2. *The current study only highlighted the structural similarities between porcine and human corneas and ocular surface. Perhaps this should be made distinct in the conclusions of the study. The proteomics and biomechanics may be distinctly different between the two species.*

Thank you for your comment. We have added this in the conclusion to clarify this situation.
